# Bone Marrow Stem Cells Added to a Hydroxyapatite Scaffold Result in Better Outcomes after Surgical Treatment of Intertrochanteric Hip Fractures

**DOI:** 10.1155/2014/451781

**Published:** 2014-05-14

**Authors:** Joao Torres, Manuel Gutierres, M. Ascenção Lopes, J. Domingos Santos, A. T. Cabral, R. Pinto, Carola van Eck

**Affiliations:** ^1^Faculty of Medicine, University of Porto, Alameda Hernani Monteiro, 4200-319 Porto, Portugal; ^2^Hospital S. Joao, Alameda Professor Hernâni Monteiro, 4200-319 Porto, Portugal; ^3^CEMUC, Faculdade de Engenharia, Universidade do Porto, Rua Doutor Roberto Frias, 4200-465 Porto, Portugal; ^4^Department of Orthopaedic Surgery, University of Pittsburgh Medical Center, 3471 Fifth Avenue, Kaufman building suite 1011, Pittsburgh, PA 15213, USA

## Abstract

*Introduction*. Intertrochanteric hip fractures occur in the proximal femur. They are very common in the elderly and are responsible for high rates of morbidity and mortality. The authors hypothesized that adding an autologous bone marrow stem cells concentrate (ABMC) to a hydroxyapatite scaffold and placing it in the fracture site would improve the outcome after surgical fixation of intertrochanteric hip fractures.* Material and Methods*. 30 patients were randomly selected and divided into 2 groups of 15 patients, to receive either the scaffold enriched with the ABMC (Group A) during the surgical procedure, or fracture fixation alone (Group B).* Results*. There was a statistically significant difference in favor of group A at days 30, 60, and 90 for Harris Hip Scores (HHS), at days 30 and 60 for VAS pain scales, for bedridden period and time taken to start partial and total weight bearing (*P* < 0.05).* Discussion*. These results show a significant benefit of adding a bone marrow enriched scaffold to surgical fixation in intertrochanteric hip fractures, which can significantly reduce the associated morbidity and mortality rates.* Conclusion*. Bone marrow stem cells added to a hydroxyapatite scaffold result in better outcomes after surgical treatment of intertrochanteric hip fractures.

## 1. Introduction

Intertrochanteric fractures occur in the region between the greater and lesser trochanters of the proximal femur. They account for nearly 50% of all fractures of the proximal femur. They are more common in women, with an average patient age of 66 to 76 years. [1–6] These fractures are associated with a high percentage of comorbidities, increased dependency in activities of daily living, other osteoporosis-related fractures, and mortality rates that can be as high as 30% in the first year after fracture, mainly related to long bedridden periods [7–23]. The treatment is almost always surgical. The two most commonly used options are the dynamic hip screw (DHS) and the intramedullary nail (IMN). Despite a trend towards using the IMN in the last years, most studies have shown no difference in results between both implants, with the exception of unstable fractures which appear to show best results when treated with IMN. Fixation failure is the most common complication with both implants, as high as 20%; it usually results from varus collapse of the proximal fragment with cutout of the lag screw from the femoral head [24–27]. Improvements in fracture fixation techniques have failed to improve this complication rate. Therefore, an effort towards improving the biologic environment of fracture healing is being pursued [28–32]. According to the diamond concept [33, 34], four factors are essential for new bone formation, as is required when treating a fracture: osteoconductivity, osteogenesis, osteoinductivity, and mechanical stimulus. The authors have based their work on these principles by attempting to add a bone marrow enriched hydroxyapatite scaffold to the fracture site. This optimizes the use of the diamond principles as follows: osteoconductivity is provided to the fracture site by a glass reinforced hydroxyapatite composite (GRHC) in a microporous pellet formulation; osteogenesis and osteoinductivity are accomplished by adding autologous bone marrow concentrate (ABMC), rich in mesenchymal stem cells (bone precursors) and growth factors, to the inorganic bone substitute; and weight bearing as tolerated by the patient provides the necessary mechanical stimulus.

The tested hypothesis was that in conjunction with the correct surgical treatment, intertrochanteric fractures would heal faster and have improved immobilization/bedridden time when a bone marrow aspirate enriched glass reinforced hydroxyapatite composite (GRHC) is applied to the fracture site.

## 2. Material and Methods

### 2.1. Patient Selection

Thirty patients with a low energy mechanism intertrochanteric fracture were selected for the study. The study was approved by the Institution's Ethical Commission, and written informed consent was given by all patients. Pathologic fractures, reverse obliquity fractures, and fractures extending to the subtrochanteric region were excluded. Patients were randomly assigned into two groups (A and B) of 15 patients, according to age, associated pathologies, medication, dependency in activities of daily living, and FRAX score [35]. Both groups were surgically treated with a DHS, by the same team of surgeons, using the standard surgical technique [6].

### 2.2. Glass Reinforced Hydroxyapatite Composite (GRHC) Pellets

The inorganic scaffold selected is a glass reinforced hydroxyapatite composed of a modified hydroxyapatite matrix with a homogeneous dispersion of *α*- and *β*-tricalcium phosphate (TCP) secondary phases and controlled percentages of ionic species, such as sodium and fluoride, which aims to mimic the chemical composition of the mineral phase of human bone [36–41]. Previous data revealed an improved* in vitro* performance and an excellent* in vivo* osseointegration with a sustained controlled resorption of the material [42–46].

Pellets were fabricated according to the Patent WO/2010/02155919. Preparation of the pellets began with the mixing of hydroxyapatite and a bioglass (with the composition 65P_2_O_5_-15CaO-10CaF_2_-10Na_2_O, mol%) with microcrystalline cellulose, followed by an extrusion and spheronization process. Pellets were then dried at 60°C and submitted to a thermal treatment. First, pellets were heat-treated at 600°C to burn out the microcrystalline cellulose and, then, they were sintered at 1300°C for 1 h, followed by natural cooling inside the furnace.

Pellets were observed by scanning electron microscopy (SEM) equipped with an energy-dispersive X-ray spectroscopy (EDS) system (Joel JSM 35C; voyager XRMA System, Noran Instruments), to characterize the macro- and microstructure and qualitatively assess pore morphology. Porosity was determined by mercury porosimetry (Auto Pore IV 9500, Micromeritics, Aachen, Germany) which is based on the intrusion of mercury under pressure into the samples pores. Surface area was achieved by BET method. Detailed physicochemical profile of the pellets had previously been reported [47]. Results showed that pellets presented a spherical shape with a particle size range of 1000–4000 *μ*m, a microporosity of 25.3%, and a surface area of 0.0171 m^2^/g. Pellets exhibited surface rugosity and, also, microporosity, which increases surface area and is expected to favor cell adhesion. These characteristics are important for the formation of interconnecting pores between the pellets which are of critical importance for osseous ingrowth [47].

Prior to bone marrow cell seeding, pellets were sterilized by autoclaving (120°C, 20 min).

### 2.3. Preparation of the Bone Marrow Concentrate of Nucleated Cells

Human bone marrow was collected from the posterior iliac crest of the patients [48–50], under anesthesia, just before starting the fracture fixation procedure. The patients were positioned in lateral decubitus and after prepping and draping of the posterior iliac crest, bone marrow (BM) was extracted with a needle coated with heparin.

A bone marrow concentration system (BMCS), MarrowStim from Biomet, was used to achieve a rapid preparation of a concentrate of nucleated cells from a sample of bone marrow aspirate. After the extraction of the bone marrow aspirate (30 mL), the cell separator was loaded with the aspirate and centrifuged at 3200 rpm for 15 minutes. After removing the plasma from the cell separator, the portion of nucleated cell concentrate was extracted (3 mL). Flow cytometry was performed to analyze the bone marrow aspirate and concentrate for cell viability and number of total nucleated cells and hematopoietic CD34^+^ cells (Stem-Kit; Beckman Coulter, Ref IM3630).

### 2.4. Surgical Procedure

Bone marrow was only collected from patients in Group A. While performing the surgical procedure, the aspirate was concentrated by a centrifuge inside the operating room. It was then added (3 mL) to the GRHC pellets (5 g), in a metallic recipient, on the operating table, resulting in a homogenous mixture.

The standard surgical technique [6] for the treatment of these fractures with a dynamic hip screw was used. With the patient in a traction table, closed reduction of the fracture was achieved with radiological support. A lateral approach of the hip was used, with incision of the* tensor fasciae latae* and partial incision of the posterior portion of the* vastus lateralis muscle*. After a small exposition of the lateral proximal femur, drilling and tapping for the DHS were performed. The homogenous mixture of the bone marrow concentrate and the GRHC pellets was then delivered, through the screw canal, into the fracture site, using a curette. The Screw was then immediately applied, in order to prevent extravasation of the mixture from the canal. The plate was then inserted, and fixation to the femur with 4.5 mm screws was performed, without further changes from the original described technique. Patients from Group B were submitted to the traditional procedure, without bone marrow harvesting or GRHC pellets application.

### 2.5. Patient Follow-Up

All patients from both groups received the same postoperative protocol. A drain was maintained for 36–48 hours. A complete blood count (CBC) was collected on postoperative day 1, and red blood cells transfusions were applied when necessary. An anteroposterior and a lateral view hip radiograph were obtained at day 1, during the hospital stay, and at days 30, 60, and 90 during follow-up in the outpatient clinics. Harris Hip Scores and visual analog pain scales (VAS) were collected at days 30, 60, and 90. Mean bedridden period in the first month and time from the injury to the start of partial and total weight bearing were registered. No patient received specific physical therapy, except passive mobilization of the limb as tolerated.

## 3. Statistical Analyses

Statistical analysis was performed using the SPSS version 19.0 (SPSS, Chicago, IL, USA). Comparisons between groups were performed by one-way ANOVA with a post hoc Turkey test. Differences were considered statistically significant at *P* < 0.05.

## 4. Results

**Figure 1:**
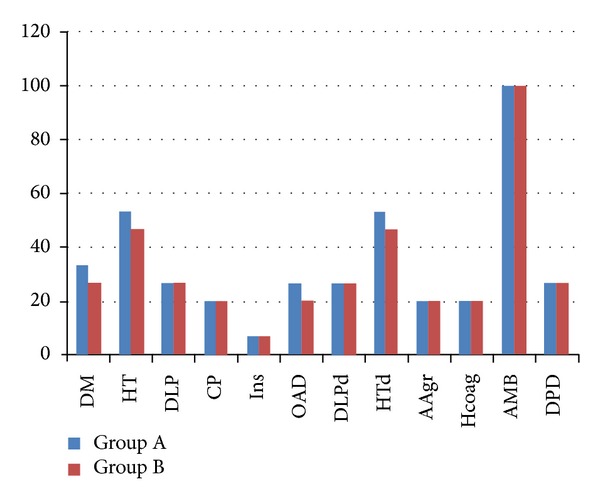
There was no statistical difference for the tested characteristics of both groups. (DM: diabetes mellitus; HT: hypertension; DLP: dyslipidemia; CP: cardiac pathology; Ins: insulin; OAD: oral antidiabetic drugs; DLPd: dyslipidemia drugs; HTd: hypertension drugs; AAgr: antiaggregants; Hcoag: hypocoagulation; AMB: ambulatory patient; DPD: dependent patient.)

Thirty patients were included in this study. The average age was 83.9 (A-84.3/B-83.5); 4 patients were males (13.3%) and 26 were females (86.7% of females in both groups). There was no statistical significant difference (*P* < 0.05) in baseline demographic characteristics between the groups for age, sex, associated pathologies (diabetes, hypertension, dyslipidemia, or cardiac pathology), medication (insulin/oral antidiabetic medication, dyslipidemia drugs, antihypertensive drugs, antiaggregants, or oral hypocoagulation), dependency in activities of daily living (ambulatory/nonambulatory and independent of/dependent on help from others), and FRAX score for the probability of hip fracture (A-17.9/B-17.6) (Figure 1).

Flow cytometry analysis (Table 1) shows the differences in cell viability of total nucleated cells, concentration of nucleated cells, concentration of CD34^+^ cells, and expected percentage of mesenchymal stem cells (MSCs), between the BM concentrate and aspirate (mean values).

**Figure 2:**
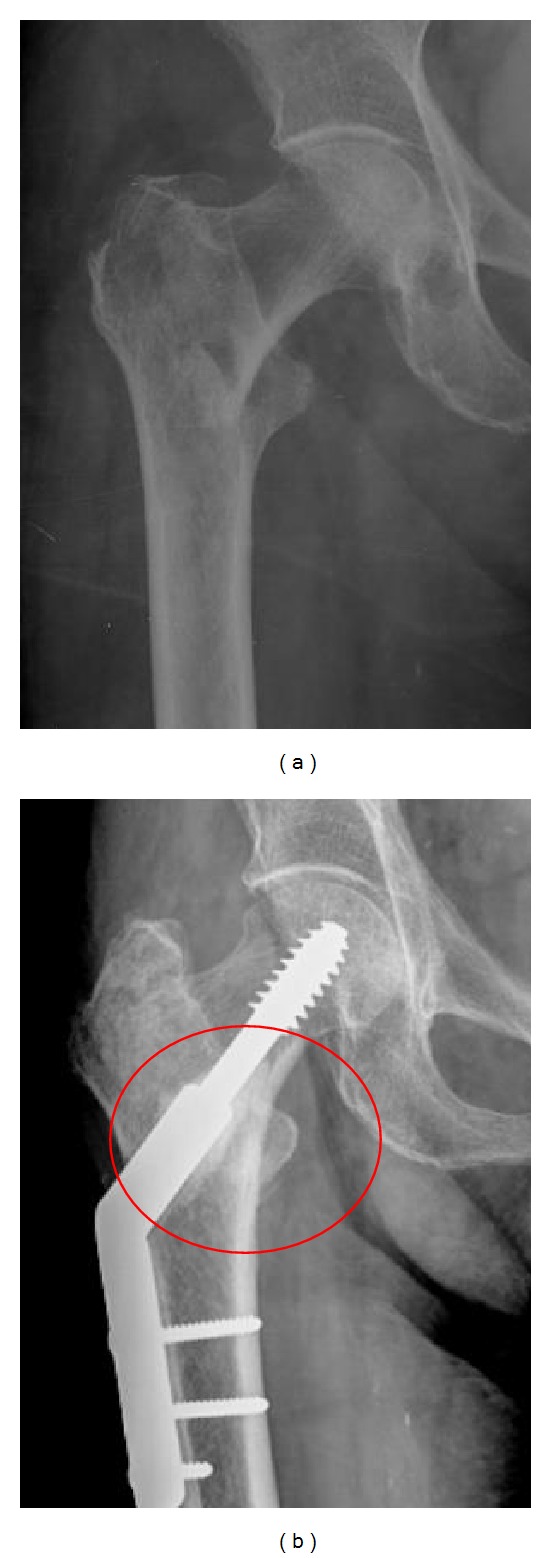
An X-Ray at 0 and 90 days of a patient from group A, showing higher density on the region where the ABMC/GRHC was added. ABMC: autologous bone marrow concentrate; GRHC: glass reinforced hydroxyapatite composite.

The hip X-rays obtained at days 1, 30, 60, and 90 showed fracture union in all cases from both groups, at the end of the study period. One case from Group B had a cutout at 60 and 90 days X-rays. In group A, an area with a higher radiological density was observed around the fracture area (Figure 2).

**Figure 3:**
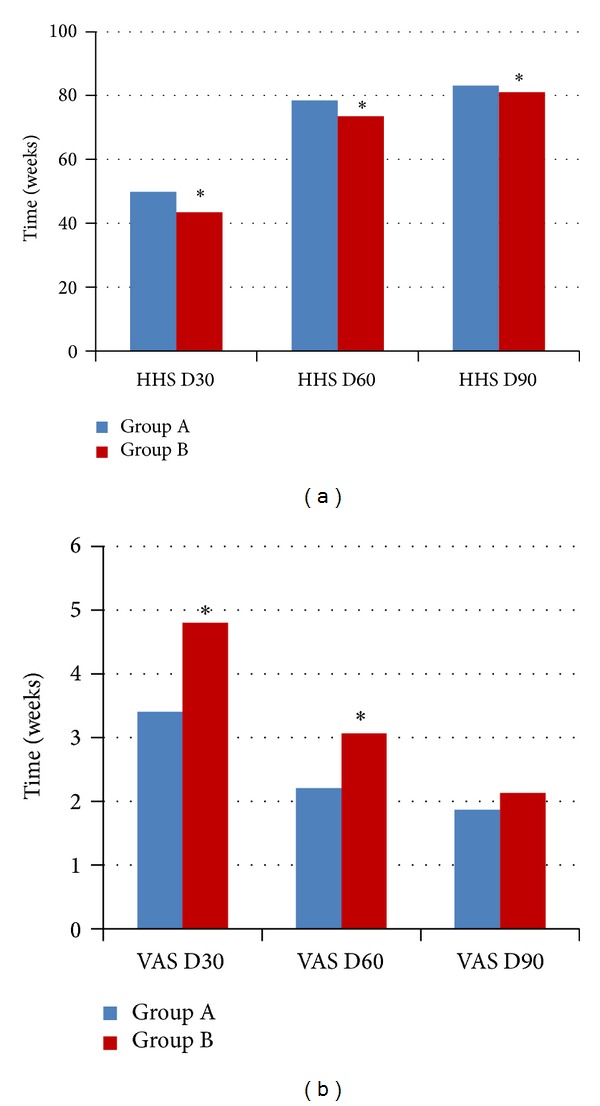
Harris Hip Score (HHS) and VAS pain scale comparison between Groups A and B at 30, 60, and 90 days (*P < 0.05).

Figure 3 shows, respectively, the comparison between both groups when considering Harris Hip Scores and visual analog pain scales (VAS) collected at days 30, 60, and 90 (HHS, 49.8 versus 43.4/78.5 versus 73.5/83.1 versus 81.1; VAS, 3.4 versus 4.8/2.2 versus 3.1/1.9 versus 2.1). Both scores showed statistically significant differences (*P* < 0.05) in the three periods, except for the VAS scale at 90 days.

**Figure 4:**
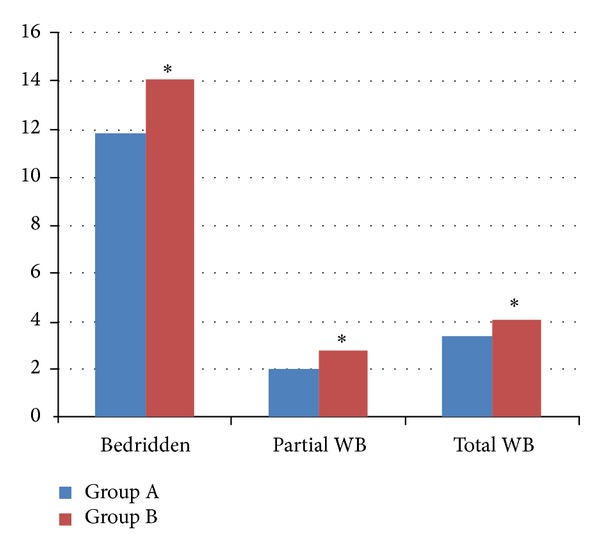
Mean bedridden period and time to partial and total weight bearing comparison between Groups A and B at 30, 60, and 90 days (*P < 0.05).

The mean bedridden period in the first month differed from 11.9 hours/day in Group A and 14.1 hours/day in Group B. Patients in Group A started partial and total weight bearing at 2.0 and 3.4 weeks (mean values), respectively. Group B patients started partial and total weight bearing at 2.8 and 4.0 weeks (mean values), respectively. Differences between both groups in these 3 variables were statistically significant (*P* < 0.05) (Figure 4).

## 5. Discussion

The present study evaluated the effectiveness of adding a bone marrow enriched hydroxyapatite scaffold to surgical fixation of intertrochanteric hip fractures. To the authors' knowledge, no previous work has been reported regarding this technique for the treatment of these kinds of fractures. Also, there is a paucity of reported data on the use of these techniques when treating acute fractures. Most of the literature reports the use of bone substitutes associated with stem cells for the treatment of nonunions or bone defects [48, 49].

Flow cytometry analysis (Table 1) showed that BM concentrate presented a significantly higher mean concentration of nucleated cells (2 × 10^7^ cell/mL) compared to that on BM aspirate (2.7 × 10^6^ cell/mL), which is expected considering the experimental protocol, as 30 mL of BM aspirate yielded 3 mL of BM concentrate. Of these, the mean number of hematopoietic CD34^+^ was 8 × 10^4^ cell/mL and 8 × 10^3^ cell/mL, respectively, in the BM concentrate and the BM aspirate. These cells are blood cell precursors and not bone tissue precursors as are mesenchymal stem cells (MSCs). The authors' large experience in quantifying this type of cells made it more accurate to measure the concentration of CD34^+^ cells. Therefore, and according to previous studies reporting that the percentage of MSCs in the nucleated cell fraction harvested from the BM is 0.01 to 0.001% [48–50], in the general population, the authors inferred that the BM concentrate contained a higher number of MSCs, about 10 times of that expected in the BM aspirate. In addition, flow cytometry analysis (Table 1) also showed that mean cell viability was found to be higher in the BM concentrate (76%) compared to that on BM aspirate (53%), most probably because the process of BM concentration eliminated a number of nonviable cells. As the same volume/mass rate was used to prepare the cell/material constructs, a better performance is anticipated with the BM concentrate/GRHC pellets.

No significant difference was evident from radiographic serial evaluation. This may be explained by the low sensitivity of this method to evaluate quantitatively new bone formation. However, there was a patient with cutout on radiographs performed at 60 and 90 days postoperatively in Group B. The authors considered two other options for evaluation of bone formation: serial DMOs or periodical micro-CTs. While the first shows low sensitivity to detect small changes in bone formation in short periods of time, the second would expose patients to unacceptable levels of radiation, precluding its use.

Harris Hip Scores and visual analog pain scales (VAS) results were better in Group A patients at 30 and 60 days (*P* < 0.05), and HHS results were slightly better at 90 days (*P* < 0.05), with no statistical difference in the VAS results. This can be explained by the probable faster consolidation of the fracture in Group A, resulting in better function and pain scores in the first weeks, an effect that probably tends to be attenuated over time.

Patients in Group A spent a smaller number of hours in bed in the first month. They also started walking earlier. Long bedridden periods and long periods without partial and total weight bearing are associated with a higher number of comorbidities and mortality rates [7–23].

Strong points of the present study are that the two groups were comparable with regard to demographic variables, including age, pathologies, medication, level of dependence, and fracture risk. However, this study also has limitations. The sample size of the present study is small. However, significant differences were found for most variables, suggesting adequate power. Second, the follow-up period of the present study is relatively short. However, the outcomes of interest to this study are only expected to be different in the first 30–60 days postoperatively.

## 6. Conclusion

The addition of a combination of a glass reinforced hydroxyapatite scaffold and a bone marrow concentrate in patients surgically treated for intertrochanteric hip fractures showed improved results in Harris Hip Scores, visual analog pain scales (VAS), bedridden periods, and time taken to start partial and total weight bearing of the affected limb.

These results show that patient quality of life is improved by the use of this combination and suggest that the high rates of morbidity and mortality associated with the treatment of patients with this type of fractures can be improved by the addition of this new scaffold.

## Figures and Tables

**Table 1:** Flow cytometry analysis of the BM concentrate and aspirate.

	Cell viability	Total of nucleated cells	CD34^+^ cells	Expected mesenchymal stem cells
BM concentrate	78% ± 0.3	2.1 × 10^7^ cell/mL ± 0.1	8.2 × 10^4^ cell/mL ± 0.3	200–2000 cell/mL
BM aspirate	51% ± 0.3	2.6 × 10^6^ cell/mL ± 0.2	8.0 × 10^3^ cell/mL ± 0.2	27–270 cell/mL

## References

[1] Abrahamsen B, van Staa T, Ariely R (2009). Excess mortality following hip fracture: a systematic epidemiological review. *Osteoporosis International*.

[2] Larsson S, Friberg S, Hansson L-I (1990). Trochanteric fractures. Mobility, complications, and mortality in 607 cases treated with the sliding-screw technique. *Clinical Orthopaedics and Related Research*.

[3] Parker MJ, Pryor GA, Myles J (2000). 11-Year results in 2,846 patients of the Peterborough Hip Fracture Project: reduced morbidity, mortality and hospital stay. *Acta Orthopaedica Scandinavica*.

[4] Rapp K, Becker C, Lamb SE, Icks A, Klenk J (2008). Hip fractures in institutionalized elderly people: incidence rates and excess mortality. *Journal of Bone and Mineral Research*.

[5] Zidén L, Wenestam C-G, Hansson-Scherman M (2008). A life-breaking event: early experiences of the consequences of a hip fracture for elderly people. *Clinical Rehabilitation*.

[6] Orthopaedic Trauma Association Classification DaOCatACSC (2007). Orthopaeidc Trauma Association. Fracture and dislocation compendium. *Journal of Orthopaedic Trauma*.

[7] Anand S, Buch K (2007). Post-discharge symptomatic thromboembotic events in hip fracture patients. *Annals of the Royal College of Surgeons of England*.

[8] Becker C, Fleischer S, Hack A (1999). Disabilities and handicaps due to hip fractures in the elderly. *Zeitschrift fur Gerontologie und Geriatrie*.

[9] Bruyere O, Brandi M-L, Burlet N (2008). Post-fracture management of patients with hip fracture: a perspective. *Current Medical Research and Opinion*.

[10] Foster MR, Heppenstall RB, Friedenberg ZB, Hozack WJ (1990). A prospective assessment of nutritional status and complications in patients with fractures of the hip. *Journal of Orthopaedic Trauma*.

[11] Freeman C, Todd C, Camilleri-Ferrante C (2002). Quality improvement for patients with hip fracture: experience from a multi-site audit. *Quality and Safety in Health Care*.

[12] Holt G, Smith R, Duncan K, Hutchison JD, Gregori A (2008). Outcome after surgery for the treatment of hip fracture in the extremely elderly. *Journal of Bone and Joint Surgery A*.

[13] Imura K, Ishii Y, Yagisawa K, Matsueda M (2000). Postoperative ambulatory level after hip fracture in the elderly predicts survival rate. *Archives of Orthopaedic and Trauma Surgery*.

[14] Kamel HK, Iqbal MA, Mogallapu R, Maas D, Hoffmann RG (2003). Time to ambulation after hip fracture surgery: relation to hospitalization outcomes. *Journals of Gerontology A Biological Sciences and Medical Sciences*.

[15] Koval KJ, Friend KD, Aharonoff GB (1996). Weight bearing after hip fracture: a prospective series of 596 geriatric hip fracture patients. *Journal of Orthopaedic Trauma*.

[16] Morrison RS, Magaziner J, McLaughlin MA (2003). The impact of post-operative pain on outcomes following hip fracture. *Pain*.

[17] Moseley AM, Sherrington C, Lord SR, Barraclough E, St George RJ, Cameron ID (2009). Mobility training after hip fracture: a randomised controlled trial. *Age and Ageing*.

[18] Oude VR, Banerjee S, Horan M (2006). Fear of falling more important than pain and depression for functional recovery after surgery for hip fracture in older people. *Psychological Medicine*.

[19] Pedersen SJ, Borgbjerg FM, Schousboe B (2008). A comprehensive hip fracture program reduces complication rates and mortality. *Journal of the American Geriatrics Society*.

[20] Perez JV, Warwick DJ, Case CP, Bannister GC (1995). Death after proximal femoral fracture: an autopsy study. *Injury*.

[21] Sherrington C, Lord SR, Herbert RD (2004). A randomized controlled trial of weight-bearing versus non-weight-bearing exercise for improving physical ability after usual care for hip fracture. *Archives of Physical Medicine and Rehabilitation*.

[22] Siu AL, Penrod JD, Boockvar KS, Koval K, Strauss E, Morrison RS (2006). Early ambulation after hip fracture: effects on function and mortality. *Archives of Internal Medicine*.

[23] Talkowski JB, Lenze EJ, Munin MC, Harrison C, Brach JS (2009). Patient participation and physical activity during rehabilitation and future functional outcomes in patients after hip fracture. *Archives of Physical Medicine and Rehabilitation*.

[24] Adams CI, Robinson CM, Court-Brown CM, McQueen MM (2001). Prospective randomized controlled trial of an intramedullary nail versus dynamic screw and plate for intertrochanteric fractures of the Femur. *Journal of Orthopaedic Trauma*.

[25] Anglen JO, Weinstein JN (2008). Nail or plate fixation of intertrochanteric hip fractures: changing pattern of practice—a review of the American Board of Orthopaedic Surgery database. *Journal of Bone and Joint Surgery A*.

[26] Chinoy MA, Parker MJ (1999). Fixed nail plates versus sliding hip systems for the treatment of trochanteric femoral fractures: a meta analysis of 14 studies. *Injury*.

[27] Ekström W, Karlsson-Thur C, Larsson S, Ragnarsson B, Alberts K-A (2007). Functional outcome in treatment of unstable trochanteric and subtrochanteric fractures with the proximal femoral nail and the Medoff sliding plate. *Journal of Orthopaedic Trauma*.

[28] Moroni A, Faldini C, Pegreffi F, Giannini S (2004). HA-coated screws decrease the incidence of fixation failure in osteoporotic trochanteric fractures. *Clinical Orthopaedics and Related Research*.

[29] Amini AR, Laurencin CT, Nukavarapu SP (2012). Bone tissue engineering: recent advances and challenges. *Critical Reviews in Biomedical Engineering*.

[30] Steinert AF, Rackwitz L, Gilbert F, Nöth U, Tuan RS (2012). Concise review: the clinical application of mesenchymal stem cells for musculoskeletal regeneration: current status and perspectives. *Stem Cells Translational Medicine*.

[31] Salem HK, Thiemermann C (2010). Mesenchymal stromal cells: current understanding and clinical status. *Stem Cells*.

[32] McGonagle D, English A, Jones EA (2007). (iii) The relevance of mesenchymal stem cells *in vivo* for future orthopaedic strategies aimed at fracture repair. *Current Orthopaedics*.

[33] Giannoudis PV, Einhorn TA, Marsh D (2007). Fracture healing: the diamond concept. *Injury*.

[34] Giannoudis PV, Einhorn TA, Schmidmaier G, Marsh D (2008). The diamond concept-open questions. *Injury*.

[35] Kanis JA (2008). FRAXtrade mark and the assessment of fracture probability in men and women from the UK. *Osteoporosis International*.

[36] Hussain NS, Lopes MA, Maurício MC, Ali N, Fernandes MH, Santos JD, Jackson MJ, Ahmed W (2006). Bonelike graft for bone regenerative applications. *Surface Engineered Surgical Tools and Medical Devices*.

[37] Lopes MA, Monteiro FJ, Santos JD (1999). Glass-reinforced hydroxyapatite composites: fracture toughness and hardness dependence on microstructural characteristics. *Biomaterials*.

[38] Lopes MA, Silva RF, Monteiro FJ, Santos JD (2000). Microstructural dependence of Young’s and shear moduli of P2O5 glass reinforced hydroxyapatite for biomedical applications. *Biomaterials*.

[39] Santos JD, Hastings GW, Knowles JC

[40] Santos JD, Lopes MA, Silva MA

[41] Santos JD, Lopes MA, Silva MA

[42] Lopes MA, Santos JD, Monteiro FJ (2001). Push-out testing and histological evaluation of glass reinforced hydroxyapatite composites implanted in the tibia of rabbits. *Journal of Biomedical Materials Research*.

[43] Gutierres M, Lopes MA, Sooraj Hussain N (2008). Bone ingrowth in macroporous Bonelike® for orthopaedic applications. *Acta Biomaterialia*.

[44] Gutierres M, Dias AG, Lopes MA (2007). Opening wedge high tibial osteotomy using 3D biomodelling Bonelike® macroporous structures: case report. *Journal of Materials Science: Materials in Medicine*.

[45] Gutierres M, Hussain NS, Lopes MA (2006). Histological and scanning electron microscopy analyses of bone/implant interface using the novel Bonelike® synthetic bone graft. *Journal of Orthopaedic Research*.

[46] Gutierres M, Hussain NS, Afonso A (2005). Biological behaviour of Bonelike® graft implanted in the tibia of humans. *Key Engineering Materials*.

[47] Cortez PP, Atayde LM, Silva MA (2011). Characterization and preliminary *in vivo* evaluation of a novel modified hydroxyapatite produced by extrusion and spheronization techniques. *Journal of Biomedical Materials Research B Applied Biomaterials*.

[48] Hendrich C, Engelmaier F, Waertel G, Krebs R, Jäger M (2009). Safety of autologous bone marrow aspiration concentrate transplantation: initial experiences in 101 patients. *Orthopedic Reviews*.

[49] Jäger M, Herten M, Fochtmann U (2011). Bridging the gap: bone marrow aspiration concentrate reduces autologous bone grafting in osseous defects. *Journal of Orthopaedic Research*.

[50] Chang Y-J, Shih DT-B, Tseng C-P, Hsieh T-B, Lee D-C, Hwang S-M (2006). Disparate mesenchyme-lineage tendencies in mesenchymal stem cells from human bone marrow and umbilical cord blood. *Stem Cells*.

